# The Principles and Practice of Surgery, Illustrated by 316 Engravings on Wood

**Published:** 1852-12

**Authors:** 


*The Principles and Practice of Surgery, illustrated by* 316 *engravings on wood.* By William Pirrie, F. R. S. E., Regius Professor of Surgery in the Marischall College and University of Aberdeen; Surgeon to the Royal Infirmary, etc., etc. Edited, with additions, by John Neill, M. D., etc. Philadelphia: Blanchard & Lea. 1852.

Without conceding to this volume any special, or extraordinary merit, we are content to say that it is equal to any of the many text-books upon surgery which have been hitherto published. The assertion of the American editor, Dr. John Neill, that "the want of a suitable *text-book* on surgery has been *constantly* felt by those who are called upon to recommend to students a guide to their surgical studies" is not altogether true. "Text-books" upon surgery, and tolerably good ones too, are not remarkably scarce at the present day. But we imagine that if the number of such books was multiplied fourfold, the "want" which some teachers experience would not be supplied: since it is the incompleteness of these "systems" which has always occasioned dissatisfaction, and yet this incompleteness is inseparable from the plan of a text-book — it is not remedied in the present instance and we suppose it never will be.

The book is good in its way. It is well written, well arranged, well digested; it is also well printed and well bound up. The American editor's notes are also well enough, and numerous enough. It is our private opinion, however, that this business of adding notes is a "bore" to all parties concerned — to the publishers, to the readers and possibly to the *editors* themselves; and that if these latter gentlemen fully understood that "we," "the people," for whose sole benefit they kindly labor, would consent to their writing their names on the backs of all these volumes, without the trouble of writing a word within, they would be very grateful.

We do not feel authorized to speak for others in a matter of so much delicacy, but we shall repeat what we have once or twice before intimated for ourselves — we shall be happy to see any man's name on the back of our books whom the gentlemanly publishers see fit to place there, whether it be the name of the author or of the editor, or of both. We can, we think, appreciate the propriety of placing the name of some respectable American gentleman under that of the foreign author — as if to say, sirs, I know this man and countenance him — I make myself responsible for him. It is necessary almost, that some men, or a few men, should devote themselves to this duty of serving as ushers, but then let their services end with the business of introduction. We do not wish them to annoy us afterward in our *tête à tête* by impertinent interruptions: indeed we wish them to leave the room. For sale by Derby & Co. F. H. H.

